# Immunostaining of βA-Activin and Follistatin Is Decreased in HPV(+) Cervical Pre-Neoplastic and Neoplastic Lesions

**DOI:** 10.3390/v15051031

**Published:** 2023-04-22

**Authors:** Victor Jesus Huaringa Payano, Lara Verônica de Araújo Lopes, Larissa Rodrigues Peixoto, Keila Alves da Silva, Tania Maria Ortiga-Carvalho, Alexandre Tafuri, Annamaria Ravara Vago, Enrrico Bloise

**Affiliations:** 1 Laboratório de Patogênese Molecular, Departamento de Morfologia, Universidade Federal de Minas Gerais, Belo Horizonte 31270-910, MG, Brazil; 2 Laboratório de Endocrinologia Translacional, Instituto de Biofísica Carlos Chagas Filho, Universidade Federal do Rio de Janeiro, Rio de Janeiro 21941-902, RJ, Brazil; 3 Laboratório de Anatomia Patológica Tafuri, Belo Horizonte 30170-133, MG, Brazil

**Keywords:** βA-activin, follistatin, activin, inhibin, cervical intraepithelial neoplasia (CIN), squamous cell carcinoma (SCC), human papillomavirus (HPV), HPV genotyping, p16

## Abstract

The activin–follistatin system regulates several cellular processes, including differentiation and tumorigenesis. We hypothesized that the immunostaining of βA-activin and follistatin varies in neoplastic cervical lesions. Cervical paraffin-embedded tissues from 162 patients sorted in control (*n* = 15), cervical intraepithelial neoplasia (CIN) grade 1 (*n* = 38), CIN2 (*n* = 37), CIN3 (*n* = 39), and squamous cell carcinoma (SCC; *n* = 33) groups were examined for βA-activin and follistatin immunostaining. Human papillomavirus (HPV) detection and genotyping were performed by PCR and immunohistochemistry. Sixteen samples were inconclusive for HPV detection. In total, 93% of the specimens exhibited HPV positivity, which increased with patient age. The most detected high-risk (HR)-HPV type was HPV16 (41.2%) followed by HPV18 (16%). The immunostaining of cytoplasmatic βA-activin and follistatin was higher than nuclear immunostaining in all cervical epithelium layers of the CIN1, CIN2, CIN3, and SCC groups. A significant decrease (*p* < 0.05) in the cytoplasmic and nuclear immunostaining of βA-activin was detected in all cervical epithelial layers from the control to the CIN1, CIN2, CIN3, and SCC groups. Only nuclear follistatin immunostaining exhibited a significant reduction (*p* < 0.05) in specific epithelial layers of cervical tissues from CIN1, CIN2, CIN3, and SCC compared to the control. Decreased immunostaining of cervical βA-activin and follistatin at specific stages of CIN progression suggests that the activin–follistatin system participates in the loss of the differentiation control of pre-neoplastic and neoplastic cervical specimens predominantly positive for HPV.

## 1. Introduction

Cervical cancer is the fourth most common type of cancer among women, resulting in an estimated mortality rate (2018–2020) of 311,000 women worldwide [1]. The development of cervical cancer is strongly related to high-risk (HR) Human Papillomavirus (HPV) infection [2,3,4]. However, other endogenous and exogenous risk factors contribute to advancing cervical cancer. Examples of these exogenous factors comprise sexual precocity, a diversity of sexual partners, the presence of co-infection, the socio-economic profile [3,5], and HPV typing (high versus low risk) [6].

HPV is a small non-enveloped circular DNA virus classified into two main categories: HR-HPVs, which include the HPV types 16, 18, 31, 33, 35, 39, 45, 51, 52, 56, 58, 59, 66, and 68, with HPV16 and HPV18 being the most prevalent and oncogenic ones, and low-risk (LR)-HPVs, which include the HPV types 6, 11, 40, 42, 43, 54, 61, 70, 72, and 81, that are primarily associated with the development of benign genital and skin wart lesions [7,8]. The HR-HPV genome has two regions that encode important proteins that mediate HPV carcinogenesis and cervical cell viral entry: the early region, encoding the early *E1, E2, E4, E5, E6,* and *E7* genes*,* and the long control region (LCR), encoding the L1 and L2 capsid-binding proteins [9]. HR-HPV types act via the continuous expression of the oncoproteins E6 and E7 to modulate intricate pathways involved in host immune responses that allow persistent cervical infection and cellular neoplastic transformation [4]. More specifically, the E6 and E7 oncoproteins are implicated in uncontrolled cell proliferation as they inactivate the tumor suppressor proteins p53 and pRB, respectively. The HR-HPV E6 oncoprotein generates a complex with the E3 ubiquitin ligase E6-associated protein (E6AP) to degrade p53 by polyubiquitination. However, the E7 oncoprotein, via the binding of an ordered zinc finger motif to pRb at the pRB/E2F suppressor complex, releases E2F (a transcription factor) and enables the HPV-infected cells to re-enter mitosis and proliferate [9,10]. In addition, some *E6* spliced isoforms (E6 *) have been hypothesized to play a role in HPV-induced carcinogenesis [11].

Women are frequently exposed to mucosal HPV infections during their first sexual intercourses; however, most of the of sexually active women infected by HPV during early adulthood (>90%) will resolve the HPV infection via actions of the host immune system- within 2 years. If persistent, cervical HPV will lead to the development of pre-neoplastic or neoplastic lesions of the cervix [4]. The mechanisms of HPV infection include the access of the virus, through micro-abrasions of the epithelium, to the mitotically competent cells of the cervical squamous basal epithelium. This is followed by the binding of the HPV-major capsid protein L1, as well as of the minor capsid protein, L2, to the cell-surface heparan sulfate proteoglycans (HSPGs) of the cervical basal cells, resulting in L1 and L2 conformational changes and internalization of the viral capsid, episomal viral genome replication, and control of the cell replication machinery to promote viral genome duplication [8,12,13,14,15]. Infected cervical basal cells will undergo abnormal differentiation and delayed apoptosis and migrate into the cervix epithelium’s intermediate and superficial layers, resulting in an altered cervical epithelium phenotype [14,16]. In this connection, cervical lesions induced by HPV can be classified according to established histopathological criteria as low-grade intraepithelial lesion (LSIL) or high-grade intraepithelial lesion (HSIL). LSIL is also referred to as cervical intraepithelial neoplasia (CIN) of grade 1 (CIN-1), whereas HSIL is subdivided into CIN-2 and CIN-3 [8,17,18,19]. If untreated, high-grade CIN-3 lesions can evolve into invasive squamous cell carcinoma (SCC) [20,21]. Of importance, cervical cancer may be prevented by regular screenings of Pap smears with clinically validated HR-HPV tests, vaccination with a nonavalent HPV vaccine, and the clinical management of advanced cancers [22].

Activins are growth factors belonging to the transforming growth factor (TGF)-β superfamily, which is extensively implicated in the onset of several proliferative disorders. Activins, which are also considered to be peptide hormones or cytokines, are polypeptide dimers bound by a disulfide bridge. There are five types of activins formed by two β-dimers each, namely, activin A, formed by two βA subunits (βA-βA), activin B (βB-βB), activin AB (βA-βB), activin C (βC-βC), and activin E (βE-βE). Importantly, activin A is the isoform of greater physiological importance in humans [23]. Activins initiate their signaling by binding to transmembrane serine/threonine kinase receptors, the activin receptor-type II (ActRIIA or ActRIIB), with the subsequent recruitment, phosphorylation, and activation of another activin receptor, ActRI [24]. The recruitment and phosphorylation of intracellular proteins known as Small Mothers Against Decapentaplegic (SMADs) 2 and 3 follow. An auxiliary SMAD 4 is incorporated into the complex, which translocates into the nucleus and regulates targeted gene transcription [23,25,26].

Follistatin and inhibins are two major regulatory elements preventing activins from functionally activating their transmembrane receptors [23]. Follistatin is a single-chain glycosylated protein that blocks activin’s ActRII and ActRI binding sites [27,28]. In contrast, inhibins are protein dimers formed by two subunits, one α and one β subunit (βA or βB), which generate the inhibin A (α-βA) or inhibin B (α-βB) isoforms [29,30]. Inhibins functionally block activins by binding their α-subunit to the betaglycan–inhibin receptor, with concomitant binding of its β-subunit (A or B) to an ActRII receptor. This interaction prevents activins from binding and activating ActRI [29].

Activins can be secreted by several cell types and have distinct actions in different cells and tissues [25], including the kidney [31], liver [32], and the reproductive system [23], among others. The loss or enhancement of activin A functional expression can impact the tissue microenvironment and alter the proliferative/differentiative states of different cell types, leading to diverse proliferative disorders [23]. Similarly, the disruption of functional follistatin can affect the proliferation of tumor cells and plays an essential role in the progression of malignant diseases [33,34]. Therefore, the active interaction of the activin–follistatin system is related to the angiogenesis, metastasis, and proliferation of solid tumors. This interaction can induce or inhibit proliferation and differentiation depending on the cell type [35].

To date, knowledge about the role of the activin–follistatin system in the progression of cervical cancer is limited [36]. It has been demonstrated that the altered immunostaining of the βA and βB subunits is related to CIN lesion progression [37]. However, the expression profile of the βA-subunit and follistatin during CIN lesion progression and its relation to HPV infection have not been determined. We hypothesized that the immunostaining of βA-activin and follistatin could potentially vary in a cell type-dependent manner in pre-neoplastic (CIN1-3) and neoplastic (SCC) cervical lesions. In this study, we evaluated the immunolocalization profile of components of the activin–follistatin system (the βA-subunit and follistatin) in cervical tissues exhibiting different stages of pre-neoplastic/neoplastic development and in the context of HPV infection. Knowledge about the expression profile of the activin–follistatin system during CIN progression, predominantly positive for HPV, can positively impact strategies for the diagnosis and control of cervical cancer.

## 2. Materials and Methods

### 2.1. Patients

This is a retrospective cohort study that includes 162 cervical biopsies of paraffin-embedded tissues (PETs) selected from the archival tissue bank of a large anatomical and histopathological laboratory analysis (located at Belo Horizonte, MG, Brazil). Histopathological diagnoses were performed between 2004 and 2006 (therefore the population included in our cohort did not receive HPV vaccination). Cervical biopsies were sorted into five different study groups. The control group consisted of tissues with no cervical lesions; *n* = 15. LSIL was designated as CIN-1 (*n* = 38). HSIL lesions were subdivided in two groups, CIN-2 (*n* = 37) and CIN-3 (*n* = 39), whereas the neoplastic cervical lesions were SCC (*n* = 33). Of importance, since the minimum age range of the HSIL cases enrolled in our cohort was 17 years and the CIN2 and CIN3 terminology is preferred in young women (aged < 30 years) due to higher regression rates in the former [19], we subdivided our HSIL cases into the CIN2 and CIN3 groups.

### 2.2. Histopathological and Immunohistochemistry Evaluations

PET-cervical tissues were cut into 5 μm serial cross-sections and stained with H&E (Sigma–Aldrich, São Paulo, SP, Brazil) for histopathological analysis or subjected to immunohistochemistry analysis for βA-activin or follistatin using protocols previously described [23,38,39] with minor adaptations.

Histopathological diagnosis of cervical lesions and SCC was revised by three experienced pathologists, blinded to groups, employing established criteria [40]. Three random cervical sections per patient were evaluated. The CIN-1 morphological aspects investigated were nuclear enlargement, hyperchromasia and the loss of cell polarity of the proliferative layer in up to 1/3 of the epithelium extension, and the frequent presence of koilocytes (cells exhibiting perinuclear halo and nuclear atypia) [41]. CIN-2 abnormalities comprised nuclear enlargement and or exhibiting irregular size, hyperchromasia, coarse chromatin, and an irregular membrane in the lower two-thirds of the epithelium enriched with koilocytotic atypia and some atypical mitotic cells. CIN-3 abnormalities were the presence of immature and proliferative cells in all epithelial layers, signs of severe atypia, oversized and irregular nuclei, and the frequent presence of atypical mitotic figures, but the absence of koilocytotic atypia [3,42]. SCC was diagnosed based on the presence of cellular atypia in all epithelial layers and the invasion of the adjacent stroma, characterized by infiltrating and irregularly sized and shaped nests sheets and anastomosing cords with an increased number of mitotic cells [20,43].

For immunohistochemistry, slides were deparaffinized in xylene, rehydrated in a decreasing alcohol series, and subjected to antigen retrieval by boiling in 0.01 M sodium citrate buffer (pH 6.0) solution for 30′ in a water bath at 100 °C. After cooling at room temperature (RT), slides were blocked using a PBS (1×)/BSA (bovine serum albumin, 3%) solution for one hour (RT). This was followed by incubation at 4 °C overnight in a 1:1500 dilution of anti-βA-activin (E-1 clone: sc-166503, Santa Cruz Biotechnology, Dallas, TX, USA) or anti-follistatin (ab203131, Abcam, Cambridge, UK) primary antibodies. The next day, a non-specific reaction to endogenous peroxidase was blocked by incubating the slides with a solution of hydrogen peroxide (3%)/methanol for 30′ (RT). Next, sections were incubated with biotinylated anti-mouse secondary antibody for βA-activin (Vectastain Elite ABC HRP Kit-PK 6102, Vector Laboratories, Newark, CA, USA) or anti-rabbit secondary antibody for follistatin (Vectastain Elite ABC-HRP Kit-PK 6101, Vector Laboratories), for one hour (RT). Between each step, slides were washed three times (for 10′ each) with PBS 1×. Slides were incubated for 30 min (RT) with streptavidin–HRP (horseradish peroxidase)-(SPD-060-Spring Bioscience, Pleasanton, CA, USA) and subjected to chromogenic detection of HRP activity by 3,3′-diaminobenzidine (DAB) reagent (DAB peroxidase substrate kit, SPD-060-Spring Bioscience, Pleasanton, CA, USA). Sections were counter-stained with Harris’ haematoxylin. Primary antibody replacement with normal serum provided negative controls.

Cytoplasmatic and nuclear immunoreactivity for βA-activin and follistatin was examined in different histological layers of healthy and pathological cervical tissue by visual assessment using optic microscopy (Accu-Scope EXC-400-Path-2FTB dual viewing). The immunoreactive score (IRS) of βA-activin and follistatin (Supplementary Table S1) was calculated by multiplying the percentage of positive cells by the intensity of the positively stained area in different histological cell layers/types in each sample [44,45].

### 2.3. HPV DNA Detection

To assess the HPV infective status of the cervical biopsies, 10 µm thick paraffin-embedded sections were obtained per sample as previously described [46]. Importantly, to avoid cross-contamination, the microtome and blades used to generate tissue sections were thoroughly cleaned with xylene, ethanol (100%), and sodium hypochlorite (2.5%) solution before and after the processing of each sample [46]. The sections were then processed as previously described [46]; briefly, tissue sections were placed in 2 mL Eppendorf tubes with 1 mL of xylene at 65 °C and homogenized under light agitation three times for 10′, followed by centrifugation at 3000 rpm for 10′, after which the xylene supernatants were discarded. All samples were re-suspended in 500 µL of sterile lysis buffer solution [containing 1 μg/µL of proteinase K (Sigma Chemical Co., St. Louis, MO, USA), 100 mm of Tris-HCl (pH 8.0), 10 mm of NaCl, and 50 mm of EDTA (pH 8.0), 0.2% of SDS] and incubated in a water bath at 37 °C for 1 to 7 days until complete tissue digestion. The processed final lysate was stored at −20 °C before PCR analyses. DNA quantification was undertaken in a spectrophotometer Nanodrop-ND 1000 (Thermo Scientific^®^, São Paulo, SP, BRA). The PCR amplification of the *β-globin* gene was used to control the integrity of the extracted DNA by using a previously described hemi-nested PCR protocol [47]. Briefly, primers GH20 (5′ GAAGAGCCAAGGACAGGTAC 3′) and PC04 (3′ CCACTTGCACCTACTTCAAC 5′) were used to amplify a 268 bp DNA fragment from the *β-globin* gene using cervical human lysate as a DNA template. The PCR product obtained in the first reaction was used as a template in the second PCR amplification, performed with PC03 (5′ ACACAACTGTGTTCACTAGC 3′) and PC04 (3′ CCACTTGCACCTACTTCAAC 5′) primers, able to amplify from the *β-globin* gene a 100 bp DNA fragment internal to the DNA fragment first amplified. The employed PCR conditions (composition of the PCR mixture, reagents’ concentrations, and amplification program) are described in Supplementary Table S2. Positive controls consisted of human DNA extracted from blood samples, and the negative control consisted of sterile bi-distilled water.

HPV DNA detection was undertaken by a nested PCR protocol as described previously [48]. For the first amplification round, MY09/MY11 primers (Supplementary Table S3) were used. In contrast, for the second amplification, the primers used were GP5+/GP6+ [49] (Supplementary Table S3), able to amplify DNA fragments of 450 bp and 150 bp, respectively, from the L1 gene of the HPV genome. The reaction mixture composition, as well as the PCR amplification conditions, are described in Supplementary Table S2. All amplification products (10 µL) were analyzed by electrophoresis on 6% polyacrylamide gel stained with silver nitrate (2%).

### 2.4. HPV DNA Genotyping

HPV-positive samples were genotyped for the detection of the HR-HPVs 16, 18, 31, 33, 35, 52, and 58 using both hemi-nested PCR and conventional PCR protocols. DNA detection from HPVs 16, 18, 52, and 58 was performed by hemi-nested PCR protocols developed in our laboratory, using primers and PCR conditions previously described, with minor modifications [50]. For the first amplification step, generic primers complementary to the E6 and E7 regions of the viral genome (E6CF4 and E7CR3) were used to amplify DNA fragments of 350–390 bp from HR-HPVs 16, 18, 31, and 52 [50]. Next, the first amplification product was used as a template for the second PCR, with the generic E7CR3 primer (reverse) and type-specific (forward) primers for detecting DNA from HPV16 (16SF), HPV18 (18SF), HPV31 (31SF), and HPV52 (52SF) (Supplementary Table S4), by amplification of the DNA fragments of 149 bp, 179 bp, 200 bp, and 300 bp, respectively. A search for HPV58 DNA was performed by using a conventional PCR protocol with the E7CR3 (reverse) and 58SF (forward) primer set, which can amplify a 375 bp DNA fragment from the E6/E7 HPV58 genomic region [50]. The detection of HPV33 was performed by a touch-down PCR protocol previously described [43] with specific 33 F (forward) and 33 R (reverse) primers (Supplementary Table S4) to amplify a 455 bp DNA fragment from the E1/E2 genomic region. The detection of HPV35 was set up by a conventional PCR protocol [51] using a specific primer set, 35 F (forward) and 35 R (reverse), to amplify a 290 bp DNA fragment from the E6/E7 gene region. Supplementary Table S5 shows the PCR conditions (composition of the PCR mixture, reagents’ concentration, and amplification program) for each HPV genotyping PCR described above. All of the PCR products were analyzed by electrophoresis on 6% polyacrylamide gel stained with silver nitrate (2%).

### 2.5. P16 Immunostaining

CIN1, CIN2, and CIN3 tissue specimens that were negative for both HPV DNA and HPV DNA genotyping analysis were evaluated for p16 immunostaining, a surrogate marker for HR-HPV [52]. Tissue sections (5 µm thickness) were deparaffinized, rehydrated, and submitted to antigenic recovery using Trilogy TM^®^ (1:100 in distilled water; Cell Marque; 920P-07, Darmstadt, Germany). This step was undertaken in a two-step washing procedure enclosed in a 100 °C steamer for 30′ each, followed by 2 washes in distilled water, RT, for 5′ each. The NovoLink kit^®^ (Novocastra Laboratories Ltd., Newcastle, UK) was used for p16 immunohistochemistry. Endogenous peroxidase activity and a nonspecific protein block were undertaken by slide immersion in specific NovoLink kit^®^ solutions for 10′ each (RT). Sections were washed twice in 1×-PBS for 5′ and incubated for 1 h (RT) with p16 monoclonal primary antibody diluted in 3% 1×-PBS/BSA at 1:100 (JC8 clone: MS-889-P1, NeoMarkers, Freemont, CA, USA). After the incubation step, sections were washed 3 times in 1× PBS for 5′ (RT). Tissue sections were subsequently treated with the post primary antibody, Block-solution, for 50′ (RT), washed twice with 1× PBS for 5′ (RT), exposed to the Novolink polymer (Novocastra Laboratories Ltd., Newcastle, UK) or 30′ (RT), and re-washed (3 times) in 1× PBS for 5′ (RT). Immunoreactivity was visualized using diaminobenzidine 3,3 (DAB) chromogen. Specimens were counter-stained with Harris’ Hematoxylin (RenyLab^®^, Barbacena, MG, BRA) and evaluated by light microscopy (Accu-Scope EXC-400–Path-2FTB dual viewing). CIN specimens were deemed positive for p16 if nucleo-cytoplasmic staining in the cervical squamous epithelium was strong and negative if nucleo-cytoplasmic staining was weak/sparse. Positive controls consisted of SCC specimens that were positive for both HPV DNA and HPV DNA genotyping. Negative controls were performed by omitting the primary antibody.

### 2.6. Statistical Analysis

Data distribution was analyzed by the D’Agostino and Pearson normality test. Differences among groups for βA-activin and follistatin IRS were assessed by the Kruskal–Wallis test for every component of the cervical epithelium and stroma followed by Dunn’s test for non-parametric multiple comparisons. All statistical analyses were performed using R-statistics computer software. Data are presented as the mean ± standard error of the mean (SEM), and differences were considered significant if *p* < 0.05.

## 3. Results

### 3.1. Patient Age Characterization

The mean patient age of all groups was 39.2 ± 15.6 years (range: 17–90). As expected, the group that exhibited the highest mean age was the SCC group (50.3 ± 15.6), whereas the group with the lowest mean age was CIN1 (30.85 ± 10.38) (Table 1). The predominant age range was 17 to 35 years for 59 women (36.4%). This predominant age range (17 to 35) was observed in the control (60%), CIN1 (68.4%), and CIN2 (37.8%) groups. For CIN3, the predominant age range was 36–50 years (20.5%), whereas for SCC, the predominant age range was 36–65 years, observed in 66.6% of cancer patients (Supplementary Table S6).

### 3.2. Histopathological Characterization of Pre-Neoplastic and Neoplastic Cervical Lesions

We conducted a detailed histopathological characterization of all sample tissue specimens included in our cohort. As expected, the cervical specimens of the control group exhibited distinct histological layers with germinative cells in the basal layer and the absence of dysplasia and HPV infection signals in the stratified epithelium (Supplementary Figure S1A–C). CIN1 histological specimens exhibited a loss of polarity and the presence of nuclear atypia in the first third of the basal epithelium (Supplementary Figure S1D–F). CIN2 showed signs of maturation disturbance in the proliferative basal layer, which exhibited a loss of cell polarity and the presence of moderate cellular atypia, restricted to 2/3 of the epithelium (Supplementary Figure S1G–I). CIN3 lesion was characterized by maturation disturbance in the proliferative basal layer showing a loss of polarity and moderate to severe atypia, reaching the entire thickness of the cervical epithelium, with a consecutive loss of stratification and mild mononuclear infiltrate in the chorion. A mononuclear infiltrate was observed in the stroma adjacent to the lesion (Supplementary Figure S1J–L). Tissue samples from the SCC group showed the proliferation of atypical epithelial cells (enlarged and hyperchromatic nuclei, with dense chromatin) arranged in blocks and solid cords, in addition to areas of necrosis with inflammatory infiltrate (Supplementary Figure S1M–O). Notably, specimens from pre-neoplastic lesions exhibited mild (CIN2) to moderate (CIN1) koilocytotic atypia in superficial and intermediate cell layers, capillary proliferation, and mild infiltrate of mononuclear cells in the chorion. Importantly, the previous initial diagnosis of all cervical biopsies was confirmed after the second histopathological analysis.

### 3.3. HPV-Positive Status

To determine the prevalence of HPV infection in our cohort, we assessed the presence of HPV DNA in cervical PET specimens diagnosed with CIN1-3 and SCC. In the present study, out of the 162 initial cervical specimens, 16 samples were inconclusive for HPV detection; therefore, all HPV detection analyses were undertaken in 146 specimens of the cohort. Almost sixty percent (59.6%) of the specimens (87/146) were positive for HPV DNA. The HPV DNA positivity rate for the CIN1 group was 46.7% (14/30). In CIN2, we detected 51.5% HPV DNA positive samples (17/33). In CIN3, we found 75% HPV DNA positivity (27/36), whereas, in the SCC group, 90.6% of the samples (29/32) were positive for HPV DNA (Table 2).

Next, we genotyped HPV types more frequently associated with the progression of pre-neoplastic lesions (Table 3). We were able to genotype 15 specimens of the control group, 30 specimens of the CIN1 group, 33 specimens of the CIN2 group, 36 specimens of the CIN3 group, and 32 specimens of the SCC group, i.e., a total of 146 samples genotyped.

Furthermore, some specimens were positive for more than one HR-HPV type. As expected, there were no HR-HPV types genotyped in the control group. HPV 16 genotype was the most prevalent in 41.2% (54/131) of the genotyped samples with cervical lesions (*n* = 131; Table 3), followed by HPV 18 and HPV 31, which showed a prevalence of 16% (21/131) and 10.7% (14/131), respectively. HPV 16, 18, and 45 genotypes were present in all groups. HPV 16 was the only type that exhibited an increase in positivity throughout lesion progression, i.e., 10% positivity (*n* = 3) in CIN1, 30.3% (*n* = 10) in CIN2, 52.8% (*n* = 19) in CIN3, and 68.8% (*n* = 22) in SCC specimens.

**Figure 1:**
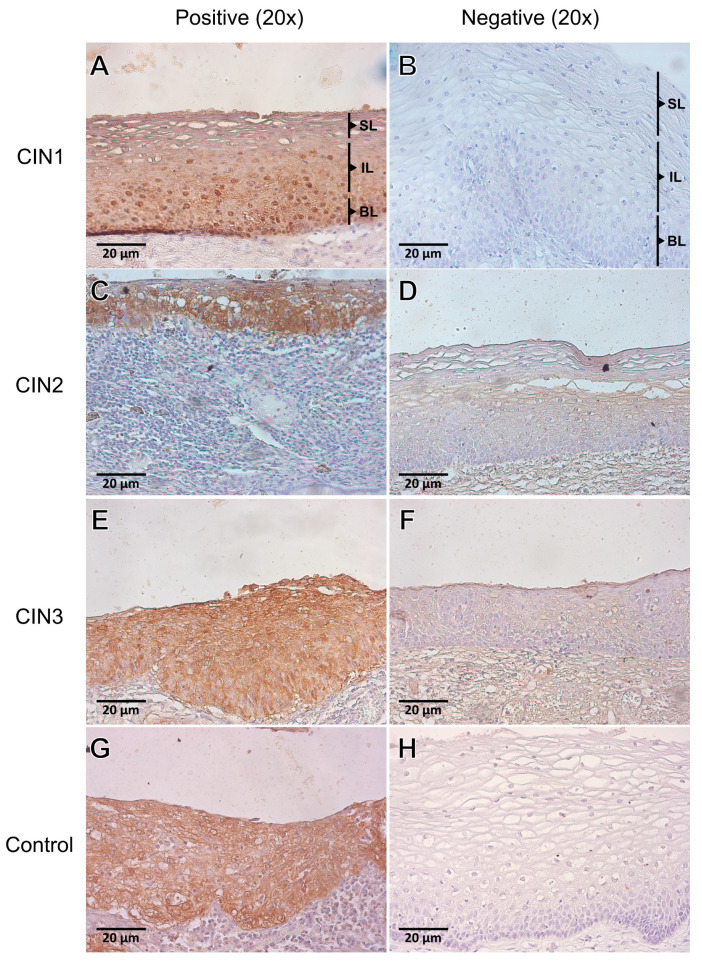
Immunolocalization of the p16-HR-HPV surrogate marker in cervical intraepithelial neoplasia (CIN) of grade 1 (*n* = 9), CIN2 (*n* = 14), and CIN3 (*n* = 6). (**A**) Diffuse cytoplasmic and nuclear p16 immunostaining was present in 1/3 of the CIN1 basal epithelium. (**C**) Representative photomicrographs of CIN2 demonstrating strong p16 staining in 2/3 of the squamous cervical epithelium. (**E**) CIN3 tissue specimens exhibited strong and diffuse p16 nucleo-cytoplasmic staining throughout the whole CIN3 lesion of the cervical epithelium. (**B**,**D**,**F**) Representative photomicrographs of CIN1, CIN2, and CIN3 specimens that were negative for p16 staining. (**G**) A CIN3-positive case for HPV DNA that served as a positive control. (**H**) A negative control of the reaction. Superficial layer (**SL**); Intermediate layer (**IL**); and Basal layer (**BL**).

We further investigated HPV positivity in CIN1 (*n* = 9), CIN2 (*n* = 14), and CIN3 (*n* = 6)-negative specimens for both HPV DNA and HPV DNA genotyping, using p16 immunoreactivity—a surrogate marker for HR-HPV [52]. Diffuse cytoplasmic and nuclear p16 immunostaining was present in 1/3 of the CIN1 basal epithelium (Figure 1A), whereas 2/3 of the CIN2 squamous cervical epithelium was stained with p16 (Figure 1C). CIN3 tissue specimens exhibited diffuse p16 nucleo-cytoplasmic staining throughout the whole CIN3 lesion of the cervical epithelium (Figure 1E).

Of the nine CIN1 cases that were double-negative for HPV in the previous analysis, six were positive for p16, two were negative, and one inconclusive. Of the 14 CIN2 cases, 11 were positive for p16 and three were negative, whereas out of the six CIN3 cases, four were positive for p16 and two were inconclusive. Next, we evaluated the overall HPV positivity rate in our cohort combining the HPV DNA, HPV DNA genotyping, and p16 immunostaining results (Table 4). We detected an overall HPV positivity of 87% (26/30) in CIN1, 91% (30/33) in CIN2, 94% (34/36) in CIN3, and 100% (32/32) in SCC.

### 3.4. Immunoreactive Scores (IRS) of βA-Activin Were Impaired in Pre-Neoplastic and Neoplastic Cervical Lesions

**Figure 2:**
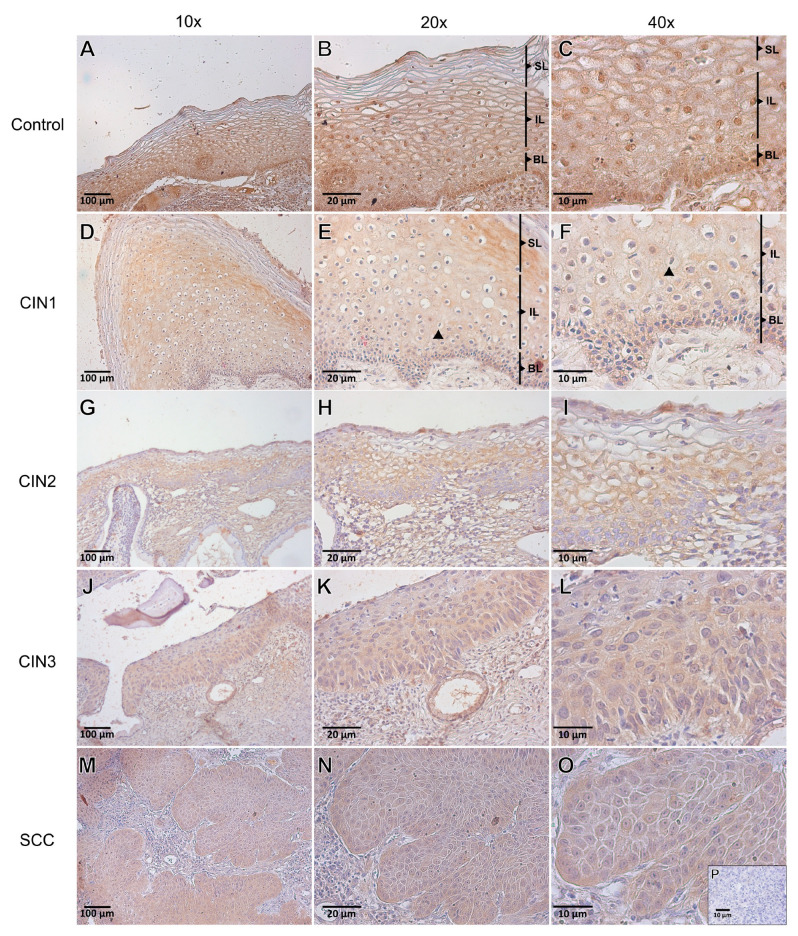
Immunolocalization of βA-activin in cervical specimens from the control (*n* = 15), cervical intraepithelial neoplasia (CIN) of grade 1 (*n* = 38), CIN2 (*n* = 37), CIN3 (*n* = 39), and invasive cervical cancer (SCC; *n* = 33) groups. (**A**–**C**) βA-activin is strongly present in the nucleus and in the cytoplasm of all epithelial and stromal cellular components in the control group. (**D**–**F**) Representative photomicrographs of the CIN1 group with moderate to strong cytoplasmic immunostaining and mild to moderate nuclear staining. (**G**–**I**) Representative photomicrographs of the CIN2 group with strong to moderate cytoplasmic staining and mild to moderate nuclear staining. (**J**–**L**) Representative photomicrographs of the CIN3 group with strong cytoplasmic immunostaining and mild to moderate nuclear staining. (**M**–**O**) Representative image of the cancer group with moderate to strong cytoplasmic immunostaining and mild nuclear immunostaining. (**P**) a negative control of the reaction. Superficial (**SL**); Intermediate (**IL**); and Basal layers (**BL**); black triangles (▲) = Mitotic figure.

Immunoreactivity studies were undertaken in 162 cervical specimens sorted in control (*n* = 15), CIN1 (*n* = 38), CIN2 (*n* = 37), CIN3 (*n* = 39), and SCC (*n* = 33) groups. βA-activin was detected in the entire cervical epithelium and stromal components in different stages of cervical cancer progression (Figure 2). Specifically, in the control group, βA-activin was detected in the cytoplasm and nucleus of all epithelial layers and stromal components (Figure 2B,C). In CIN 1, the cytoplasmic staining of the βA-activin was more intense in the basal, intermediate, and superficial layers of the epithelium (Figure 2D,F). In CIN 2, cytoplasmic immunostaining was higher in the basal and superficial layers, blood vessels, and inflammatory cells. In CIN3, the cytoplasmic immunostaining was higher in the entire cervical epithelium (indicated as CB in Figure 2K,L), as well as in blood vessels and inflammatory cells. In SCC, cytoplasmic immunostaining was observed in the neoplastic cell nests invading the stroma, as well as in blood vessels and inflammatory cells. On the other hand, in CIN1, nuclear immunostaining was evident in the basal layer (Figure 2A–L).

**Figure 3:**
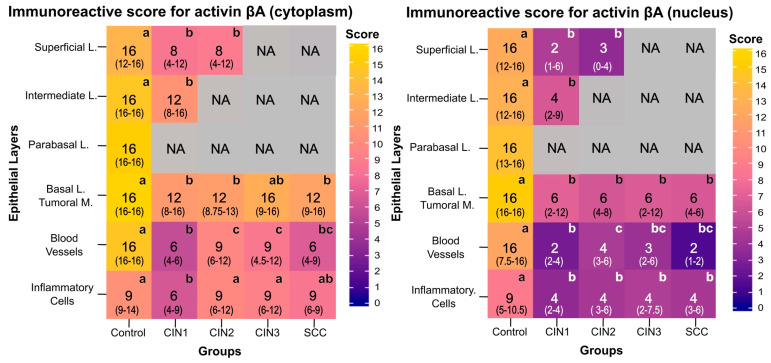
βA-activin immunoreactive scores (IRS) in the cytoplasm (left) and in the nucleus (right) in different layers of the cervical epithelium at different stages of cervical cancer progression. Data presented indicate the median and interquartile ranges (in parentheses) of the cytoplasmic and nuclear IRS in the superficial, intermediate, parabasal, and basal layers (**L**), as well as in blood vessels and inflammatory cells of the control, cervical intraepithelial neoplasia (CIN) of grade 1 (*n* = 38), CIN2 (*n* = 37), CIN3 (*n* = 39), and invasive cervical cancer (SCC; *n* = 33) groups. Darker colors indicate less βA-activin immunostaining, and lighter colors indicate more immunostaining. Group differences were assessed using the non-parametric Kruskall–Wallis test and Dunn’s multiple comparison post-test, where different letters between groups of the same layer indicate a statistically significant difference (*p* < 0.05). NA indicates unassessed values due to the loss of specific epithelium layers during neoplastic progression. In the SCC group, the tumoral mass was considered instead of the basal layer.

Next, we evaluated the IRS of βA-activin in different groups (Figure 3). The overall nuclear and cytoplasmic IRS values were similar in the control group; however, the cytoplasmic IRS values in the CIN1, CIN2, CIN3, and SCC groups were generally higher than the nuclear values. In the basal layer, the nuclear IRS values of CIN1 (*p* = 0.001), CIN2 (*p* < 0.001), CIN3 (*p* < 0.001), and SCC (*p* = 0.028) were lower than that of the control group. Similarly, the cytoplasmic IRS values of βA-activin in the CIN1 (*p* = 0.002), CIN2 (*p* = 0.003), and SCC (*p* = 0.028) groups were lower than that in the control group. In the intermediate layer of the control group, we found lower nuclear (*p* = 0.0039) and cytoplasmic (*p* = 0.0004) IRS values of βA-activin compared to those of CIN1. In the superficial layer, the nuclear IRS values of CIN1 (*p* = 0.0052) and CIN2 (*p* = 0.004) were lower than those in the control group. Accordingly, compared to the control, cytoplasmic IRS was lower in the CIN1 (*p* = 0.013) and CIN2 (*p* = 0.024) groups. In columnar epithelium, we found lower nuclear (*p* = 0.003) and cytoplasmatic βA-activin IRS values (*p* < 0.001) in CIN1 compared to the control. Similarly, CIN1 had lower cytoplasmatic IRS than that in the CIN2 (*p* < 0.001) and CIN3 (*p* = 0.0029) groups. In blood vessels, nuclear IRS in the CIN1 (*p* < 0.001), CIN2 (*p* = 0.0189), CIN3 (*p* < 0.001), and cancer (*p* < 0.001) groups was lower than that in the control group. In addition, cytoplasmic IRS was lower in the CIN1 (*p* < 0.001), CIN2 (*p* = 0.0233), CIN3 (*p* = 0.01), and cancer (*p* = 0.001) groups compared to the control. Concerning the inflammatory cell infiltrates, nuclear IRS in the CIN1 (*p* < 0.001), CIN2 (*p* = 0.03), CIN3 (*p* = 0.017), and cancer (0.0288) groups was lower than that in the control, whereas the cytoplasmic IRS of the CIN1 group had lower IRS compared to the control (*p* = 0.0047), CIN2 (*p* = 0.001), and CIN3 (*p* = 0.0192) groups.

### 3.5. Immunoreactive Scores (IRS) of Follistatin Are Impaired in Pre-Neoplastic and Neoplastic Cervical Lesions

**Figure 4:**
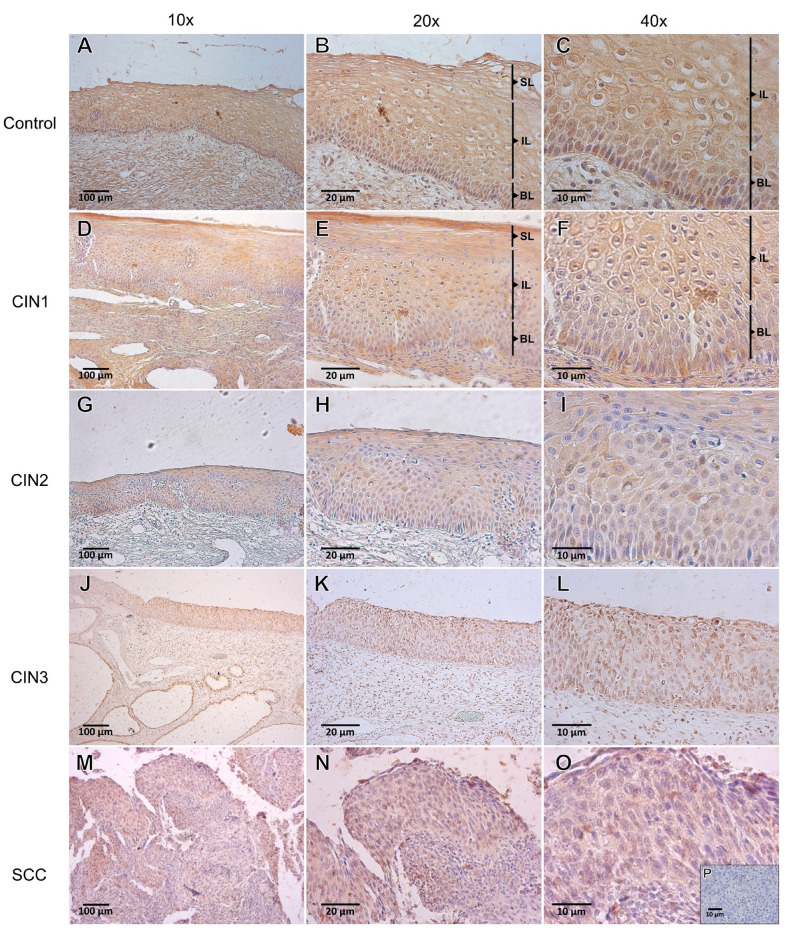
Immunolocalization of follistatin in the cervical control (*n* = 15), cervical intraepithelial neoplasia (CIN) of grade 1 (*n* = 38), CIN2 (*n* = 37), CIN3 (*n* = 39), and invasive cervical cancer (SCC; *n* = 33) groups. (**A**–**C**) Representative photomicrographs of the control group showing moderate follistatin immunostaining in the cytoplasm and in the nucleus. (**D**–**F**) Representative photomicrographs of CIN1 exhibiting strong to moderate cytoplasmic and moderate nuclear follistatin immunostaining. (**G**–**I**) Representative photomicrographs of CIN2 showing moderate cytoplasmic and mild to moderate nuclear follistatin immunostaining. (**J**–**L**) Representative photomicrographs of CIN3 with moderate cytoplasmic and nuclear follistatin immunostaining. (**M**–**O**) Representative photomicrographs of SCC showing moderate/weak cytoplasmic follistatin immunostaining. (**P**) a negative control of the reaction. Superficial (**SL**); intermediate (**IL**); and basal (**BL**) layers.

Follistatin tissue localization was detected in all cervical epithelial layers, as well as in the stromal components of cervical specimens from the control group through all stages of progression into SCC (Figure 4). In the control group, follistatin immunostaining was higher in the cytoplasm of all layers of the stratified epithelium. Nuclear follistatin labeling was higher in the parabasal, intermediate, and superficial layers of the squamous epithelium, as well as in columnar cells and stromal blood vessels. In the CIN1 group, the cytoplasmic follistatin immunolabeling was higher in the basal, intermediate layer, and blood vessels, whilst the nuclear staining was higher in the basal layer. In the CIN2 group, the cytoplasmic IRS was higher in the basal layer, columnar epithelium, and blood vessels; however, the nuclear IRS was higher only in the basal layer. In the CIN3 group, the cytoplasmic labeling was higher in the basal layer and inflammatory cells. Nuclear follistatin was higher in the basal layer. In SCC specimens, cytoplasmic follistatin immunolabeling was higher in the basal layer, while nuclear labeling was lower (Figure 4).

**Figure 5:**
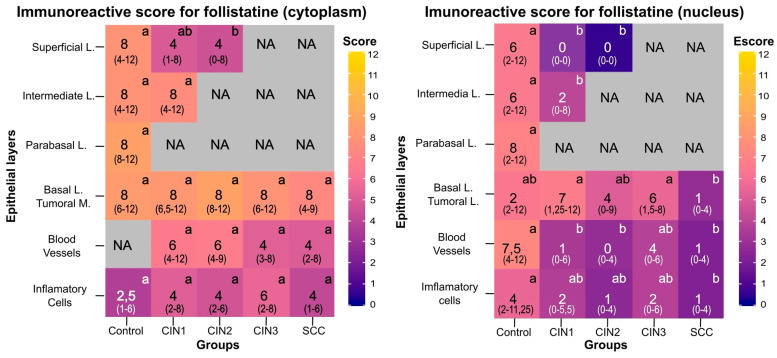
Follistatin immunoreactive scores (IRS) in the cytoplasm (left) and in the nucleus (right) in different layers of the cervical epithelium at distinct stages of the cervical cancer progression. Data presented indicate the median and interquartile ranges (in parentheses) of the cytoplasmic and nuclear IRS in the superficial, intermediate, parabasal, and basal layers (**L**), as well as in blood vessels and inflammatory cells of the control, cervical intraepithelial neoplasia (CIN) of grade 1 (*n* = 38), CIN2 (*n* = 37), CIN3 (*n* = 39), and invasive cervical cancer (SCC; *n* = 33) groups. Darker colors indicate less follistatin immunostaining, and lighter colors indicate greater immunostaining. Group differences were assessed using the non-parametric Kruskall–Wallis test and Dunn’s multiple comparison post-test, where different letters between groups of the same layer indicate a statistically significant difference (*p* < 0.05). NA indicates unassessed values due to the loss of specific epithelium layers during neoplastic progression. In the SCC group, the tumoral mass was considered instead of the basal layer.

Compared to the control group, no IRS differences in cytoplasmic and nuclear follistatin were identified in the cervical basal layer of CIN1 to CIN3 and SCC (Figure 5). However, nuclear follistatin was decreased in SCC compared to CIN1 and CIN3 (*p* < 0.001). In the intermediate layer, nuclear IRS was lower in CIN1 compared to controls (*p* = 0.045). In the superficial layer, follistatin IRS in the cytoplasm and the nucleus were lower in CIN2 compared to the control group (*p* < 0.05). In contrast, nuclear follistatin IRS in CIN1 was lower compared to controls (*p* < 0.05). In the columnar epithelium, follistatin IRS was decreased in the nucleus of CIN1 and CIN2 compared to controls (*p* = 0.002). In addition, blood vessel nuclear follistatin was decreased in CIN1, CIN2, and SCC compared to controls (*p* < 0.05). Finally, in a comparison of the nuclear follistatin IRS of inflammatory cells, the nuclear IRS of SCC (*p* = 0.037) was lower than the IRS observed in the control group.

## 4. Discussion

In the present study, we investigated the localization pattern and expression profile of βA-activin and follistatin during different stages of cervical cancer progression (from CIN1 to SCC) in unvaccinated women predominantly positive for HR-HPV infection.

We observed an increase in the mean patient age along with the progression of cervical lesions from CIN1 to SCC. These results are in agreement with previous studies reporting greater risk of cervical cancer lesions in older patients [53]. In the current study, the mean patient age was 50.3 ± 15.6 years in SCC patients. This is similar to what has been reported by Alemany and cols., 2014 [54], showing the mean age of SCC patients was 51.2 years. Together, these data demonstrate that women older than 50 years of age have higher rates of SCC diagnosis. This aging correlation is likely due to the accumulation of mutations, genomic instability, and changes in hormonal stimuli and metabolic signaling in proliferative cells of the cervical epithelium in older women, favoring the progression of CIN lesions into invasive cancer [55]. In this connection, populations with less frequent cervical cytological controls have higher rates of cervical lesions [53].

Several molecular and epidemiological studies conducted over the past 40 years elucidated that SCC development is significantly related to persistent HR-HPV infection [2,3]. In the present study, 59.9% of the analyzed cervical samples were positive for HPV DNA. In this context, higher positivity rates for HPV in cervical tissues have been reported in cervical lesions elsewhere. One study based on an HPV search using colposcopy and histopathology reported an HPV positivity rate of 96.5% [56]. In addition, a high positive rate for HPV detection (97.4%) was also verified in a cohort of tissue samples exhibiting different stages of cervical cancer disease [57].

Our cohort’s HPV positivity rate gradually increased during cervical lesion progression. Our results agree with a systematic study [58] reporting that HPV prevalence increases with CIN1–SCC lesion progression. However, other studies have reported higher HPV positivity rates starting early in CIN1 lesions [57]. Collectively, these data demonstrate that the rate of HPV positivity in cervical cancer lesions varies and that such variation may be due to the type of sample investigated (fresh frozen x formalin-fixed), the sensitivity of the HPV detection methodology, the age of patients, the severity of the cervical lesions, and the HPV vaccination status of the patients, as well as the geographical and the social regions in which these studies have been undertaken. In this context, the lower HPV DNA positivity rate found in our cohort may be related to formaldehyde exposure, the fixation protocol, and the prolonged storage of the PET samples, which could potentially contribute to the degradation of the nucleic acids [59,60]. Moreover, the HPV DNA viral load in combination with the sensitivity of the HPV DNA detection system may have contributed to the results herein obtained, especially because it has been demonstrated that the HPV viral load in LSIL remains low, whereas it increases with the progression of cervical dysplasia, therefore potentially contributing to the higher HPV DNA detection rate observed with lesion progression [61,62]. However, despite this limitation, our overall results combining HPV DNA, HPV DNA genotyping, and p16 immunohistochemistry demonstrated an increase in the HPV positivity rate along with the severity of the cervical injury, suggesting that the SCC origin in our cohort was largely due to the effects of HPV infection in early stages of cervical neoplasia. To test this hypothesis, we genotyped the most common HR-HPV types in our cohort.

The most prevalent HR-HPV types involved in the progression of intra-epithelial lesions to cervical cancer are HPVs 16, 18, and 31 [53,63,64]. In the present study, we found a similar prevalence of HPVs 16 and 18, the two most prevalent types of HPV [18,63]. In our cohort, the presence of HPV16 DNA was markedly increased as the cervical lesion progressed, similar to previous reports [56,65,66]. Importantly, the high rates of HPV positivity herein reported in the groups with cervical lesion may be largely due to the inclusion criteria of the study, which, apart from the control group, only enrolled cervical specimens diagnosed with CIN1-3 and SCC lesions–known to be induced by HPV. Furthermore, in the present study, we did not investigate the profile of βA-activin and follistatin expression in cervical tissue positive for LR-HPVs, especially in CIN1 lesions; this should be addressed in future studies.

Our study is the first to analyze βA-activin and follistatin expression profiles in the stratified epithelium layers and components of cervical human specimens. In normal cervical tissue, we detected positive immunostaining of βA-activin in all layers of the cervical epithelium, both in the nucleus and in the cytoplasm, suggesting that activin A and/or AB or inhibin A may control the proliferation and differentiation of the normal cervical stratified epithelium and stroma. There is limited information about the effect of activins, inhibins, and follistatin controlling cervical epithelial cell proliferation and differentiation. However, it has been demonstrated that activin A inhibited cell proliferation and increased the cellular volume of the PHM1 myometrial cell line [67]. Furthermore, in endometrial cells, activin A stimulated wound closure, consistent with a functional role in endometrial repair after menses [68]. Together, these data demonstrate that activins are essential regulators of proliferation and differentiation in distinct intrauterine cell types, highlighting the need for further studies identifying the precise effects of the activin–follistatin system in the human cervix.

Our study observed an overall decrease in cytoplasmic and nuclear βA-activin IRS in different stages of cervical lesion progression. This important finding suggests that lower expression of activins A and/or AB or inhibin A may be involved in cervical lesion progression as early as the initial stages of CIN1 development. It has been reported that activin A cervical fluid levels decreased after surgical removal of the lesion [69], suggesting an important role of activin A in cervical cancer pathogenesis. Importantly, activin A may regulate tumor development in a cell-type and lesion-staging manner [23]. Of particular importance, it has been demonstrated that activin A favored HPV 8–induced skin tumor progression via the action of pro-tumorigenic macrophages [70], which connect HPV infection with activin A signaling.

Our current results showing decreased βA-activin IRS in SCC specimens agree with previous findings reporting decreased activin βA and βB subunits in the epithelium of cervical adenocarcinoma compared to a healthy cervix [44]. Of importance, in this report, the labeling pattern of the βA and βB subunits in the CIN1, CIN2, and CIN3 tissues was not investigated [44]. Here, we report for the first time that the nuclear and cytoplasmic immunostaining of βA-activin in the cervical epithelium decreased from CIN1 to SCC, demonstrating a probable impairment of the signaling pathways controlled by activin A, AB and/or inhibin A, starting at CIN1 lesion formation–which could remain impaired throughout the progression of the CIN lesions into SCC. It is possible that activin A, AB, and/or inhibin A may play a central role in preventing tumorigenesis of the cervical epithelium given the essential role of activin A functioning as a tumor suppressor in the early stages of carcinogenesis in several cell types [71]. In this context, the impairment of signaling pathways stimulated by activin A, including changes in the expression and functionality of its receptors, as well as alterations in intracellular signaling pathways (SMADS) and in the expression and function of activin’s neutralizing proteins (follistatin), has the potential to alter the cell phenotypic status as well as proliferation and differentiation of cervical lesions [23].

We also identified lower cytoplasmic and nuclear immunostaining of βA-activin in the blood vessels of CIN1, CIN2, CIN3, and SCC compared to the control group. The effect of activin A regulating different aspects of angiogenesis is controversial since reports have described activin A may have either pro- [24] or anti-angiogenic effects, depending on the cell type, in different tumors [72,73]. Additionally, endothelial cells are an important source of activin A during the inflammatory response, stimulated by IL-6 or IL-1β cytokines [74]. The role of the activin–follistatin system regulating angiogenesis in human cervix lesions, as well as the knowledge of how cervical endothelial dysregulation is involved in cervical pre-neoplastic progression, requires further investigation.

Immunolocalization of βA-activin was also detected, mainly in the nucleus of mononuclear cells of the inflammatory infiltrates in the control group (which were retrieved due to clinical suspicion of cervicitis), while a decrease in βA-activin IRS was verified in subsequent stages of CIN progression. The activity of several inflammatory cells, such as macrophages, can be stimulated by activin A, which can induce a pro- or an anti-inflammatory effect depending on the cellular maturation stage [23,70]. For example, activin A can activate neutrophils via TNF-mediated induction [70]. Activin A can also inhibit the proliferation of B lymphocytes and allow the differentiation of T lymphocytes. To better discuss the biological significance of decreased βA-activin labeling in the inflammatory infiltrates during cervical lesion progression, it would be necessary to characterize the immune cell populations in the cervical lesions. This investigation should be carried out in future studies.

It is important to note that activin A is related to immune responses to viral infections. An increase in tissue activin A may be related to the inhibition of the viral replication of the hepatitis C virus, human cytomegalovirus, and Zika virus. In the chronic stages of hepatitis A and B and H1N1 infection, an increase in the local expression of activin A is related to liver lesions’ chronicity and fibrosis [70]. Here, we demonstrated that βA-activin IRS decreased in more advanced stages of cervical proliferative lesions. Activin A has been related to the inhibition of viral replication [70]. In line with the fact that in our cohort, HPV positivity increased along with the lesion severity, it is tempting to speculate that a decrease in βA-activin could be related to greater tissue positivity to HPV and, consequently, to worsening cervical lesion outcomes. Alternatively, it is possible that the decrease in βA-activin IRS might be related to molecular pathways of cervical cancer transformation and not with HPV tissue positivity per se, given the fact that activin A functions as a tumor suppressor during tumorigenesis [71] and that deranged activin A signaling is known to induce changes in the proliferative states of different cells and in distinct degrees [23,75,76,77].

With respect to follistatin, it participates in the tumorigenesis, metastasis, and angiogenesis of different tumors through its interaction with members of the TGF-β superfamily, especially activins [33,78]. In addition, it may have proliferative or proliferation inhibitory activity in different tumor types depending on the cells involved and the signaling pathways activated [79,80]. However, the understanding of the role of follistatin in the progression of cervical cancer is still quite limited [36]. Here, we observed that follistatin is expressed in all epithelial layers and stromal components of the healthy human cervix, as well as during the acquisition of the SCC neoplastic phenotype. Notwithstanding, we demonstrated that follistatin had higher nuclear immunoscore in the control group and that it decreased, in a cellular-dependent way, with progression from CIN lesions to cancer. Thus, we observed greater alterations in the follistatin nuclear immunoscore than in the cytoplasmic immunoscore. Although the biological role of nuclear follistatin is currently unknown, our data demonstrate that a decrease in nuclear follistatin immunostaining is related to the progression of cervical neoplastic injury. Furthermore, since alterations in the cytoplasmic follistatin immunostaining were not observed in this study, there may be no changes in the secretion of follistatin by cervical cells. Consequently, there may be no decrease in the magnitude of the extracellular neutralization of activin function by follistatin as cervical lesions progress into cancer.

Importantly, the decrease in the follistatin nuclear immunoscore occurred in the intermediate layers between the control group and CIN1 and in the superficial layer, between the control group and CIN1/CIN2. Although we did not observe any alterations in the follistatin immunoscore in the basal layer, changes identified in the upper epithelium may have originated in the basal layer. In cervical blood vessels, nuclear follistatin immunostaining decreased in all groups compared to the control, whereas nuclear follistatin IRS in inflammatory cells decreased in the SCC group. Follistatin is secreted by endothelial cells when stimulated by pro-inflammatory cytokines [74]. Therefore, our results indicate that nuclear follistatin could be related to angiogenesis and vascular function during cervical lesion progression [81,82].

## 5. Conclusions

We conclude that HPV16 was most prevalent in the cervical lesions of the unvaccinated population herein investigated and that βA-activin and follistatin IRS decrease in pre-neoplastic and neoplastic diseases of the human cervix, predominantly positive for HPV. Since great effort is currently being undertaken to develop therapeutic agents that modulate activin signaling [23], understanding the role of the activin–follistatin system during cervical carcinogenesis may represent future avenues of prevention and treatment of cervical pre-cancerous and cancerous disorders. Nevertheless, future studies are required to investigate the expression profiles of βB and α-inhibin subunits as well as activin and inhibin receptors and their relation to HPV infection during the acquisition of the cervical neoplastic phenotype.

## Figures and Tables

**Table 1:** Age comparison of patients enrolled in distinct groups.

	Control (*n* = 15)	CIN1 (*n* = 38)	CIN2 (*n* = 37)	CIN3 (*n* = 39)	SCC (*n* = 33)	Total (*n* = 162)
**Age**							
Mean ± SEM	36.9 ± 17.02	30.8 ± 10.38	37.6 ± 15.15	37.9 ± 10.6	50.3 ± 15.55	39.0 ± 15.47
Minimum	17	19	17	19	20	17
Maximum	65	68	81	51	90	90

HPV: human papillomavirus; CIN: cervical intraepithelial neoplasia; SCC: squamous cell carcinoma.

**Table 2:** Detection of HPV DNA in cervical specimens by nested PCR.

	Control (*n* = 15)	CIN1 (*n* = 30)	CIN2 (*n* = 33)	CIN3 (*n* = 36)	SCC (*n* = 32)	Total (*n* = 146)
*HPV*	Number of HPV (+) specimens within each group					
	(∼ % of positive specimens within each group					
Positive	0	14	17	27	29	87
	(0%)	(46.7%)	(51.5%)	(75%)	(90.6%)	(59.6%)
Negative	15	16	16	9	3	59
	(100%)	(53.3%)	(48.5%)	(25%)	(9.4%)	(40.4%)
Total	15	30	33	36	32	146
	(10.3%)	(20.5%)	(22.6%)	(24.7%)	(21.9%)	(100%)

HPV: Human Papillomavirus; CIN: Cervical Intraepithelial Neoplasia; SCC: Squamous Cell Carcinoma; *n*: number of samples.

**Table 3:** HPV DNA genotyping in cervical proliferative lesions by hemi-nested PCR and conventional PCR (the control group (*n* = 15) was all negative for HPV DNA genotyping and was therefore not included in this table).

Groups	CIN 1 (*n* = 30)	CIN 2 (*n* = 33)	CIN 3 (*n* = 36)	SCC (*n* = 32)	Total (*n* = 131)
**HR-HPV type**	Number of + specimens for each HPV genotype within each group				
	∼ % of positivity (compared to the n within each column)				
*HPV 16*	3	10	19	22	54
	10.0%	30.3%	52.8%	68.8%	41.2%
*HPV 18*	5	1	10	5	21
	16.7%	3%	27.8%	15.6%	16.0%
*HPV 31*	8	-	1	5	14
	26.7%	-	2.8%	15.6%	10.7%
*HPV 33*	3	-	-	-	3
	10.0%	-	-	-	2.3%
*HPV 35*	2	1	-	2	5
	6.7%	3%	-	6.3%	3.8%
*HPV 45*	5	3	2	2	12
	16.7%	9.1%	5.6%	6.3%	9.2%
*HPV 52*	3	4	-	4	11
	10.0%	12.1%	-	12.5%	8.4%
*HPV 58*	1	-	-	-	1
	3.3%	-	-	-	0.8%

HR-HPV: high-risk human papillomavirus; CIN: cervical intraepithelial neoplasia; SCC: squamous cell carcinoma.

**Table 4:** Detection of the overall HPV status in cervical proliferative lesions, combining HPV DNA, HPV DNA genotyping (that were previously negative for HPV DNA detection), and p16-positive specimens (that were previously negative for both HPV DNA and HPV DNA genotyping analysis).

		CIN1 (*n* = 30)	CIN2 (*n* = 33)	CIN3 (*n* = 36)	SCC (*n* = 32)	Total (*n* = 131)
HPV Positivity	HPV DNA	14	17	27	29	87
	HPV DNA Genotyping	6	2	3	3	14
	p16	6	11	4	-	21
	Total (∼%)	26 (87%)	30 (91%)	34 (94%)	32 (100%)	122 (93%)

HPV: Human Papillomavirus; CIN: Cervical Intraepithelial Neoplasia; SCC: Squamous Cell Carcinoma; *n*: number of samples.

## Data Availability

The data that support the findings of this study are available from the corresponding author upon reasonable request.

## Supplementary Materials

The following supporting information can be downloaded at: https://www.mdpi.com/article/10.3390/v15051031/s1. Figure S1: Histopathological assessment of the control, cervical intraepithelial neoplasia (CIN) of grade 1 (CIN1), CIN2, CIN3, and squamous cell carcinoma (SCC) groups; Table S1: Immunoreactive scoring (IRS) of βA-activin and follistatin; Table S2: PCR protocols and conditions used for the detection of DNA HPV; Table S3: Nucleotide sequences of the primers used for PCR detection of DNA HPV; Table S4: Nucleotide sequence of general and type-specific primers used for genotyping high-risk HPVs; Table S5: PCR protocols and conditions used for the high-risk (HR)-HPV genotyping by PCR; Table S6: Age range of the patients included in the study.
