# Supplementary material for: Immunostaining of βA-Activin and Follistatin Is Decreased in HPV(+) Cervical Pre-Neoplastic and Neoplastic Lesions
Source: Viruses. 2023 Apr 22;15(5):1031. doi: 10.3390/v15051031 (PMC10223611; doi:10.3390/v15051031)
Supplement: Supplementary file 1 [file viruses-15-01031-s001.zip › viruses-2344010-supplementary.pdf]

Supplementary Data

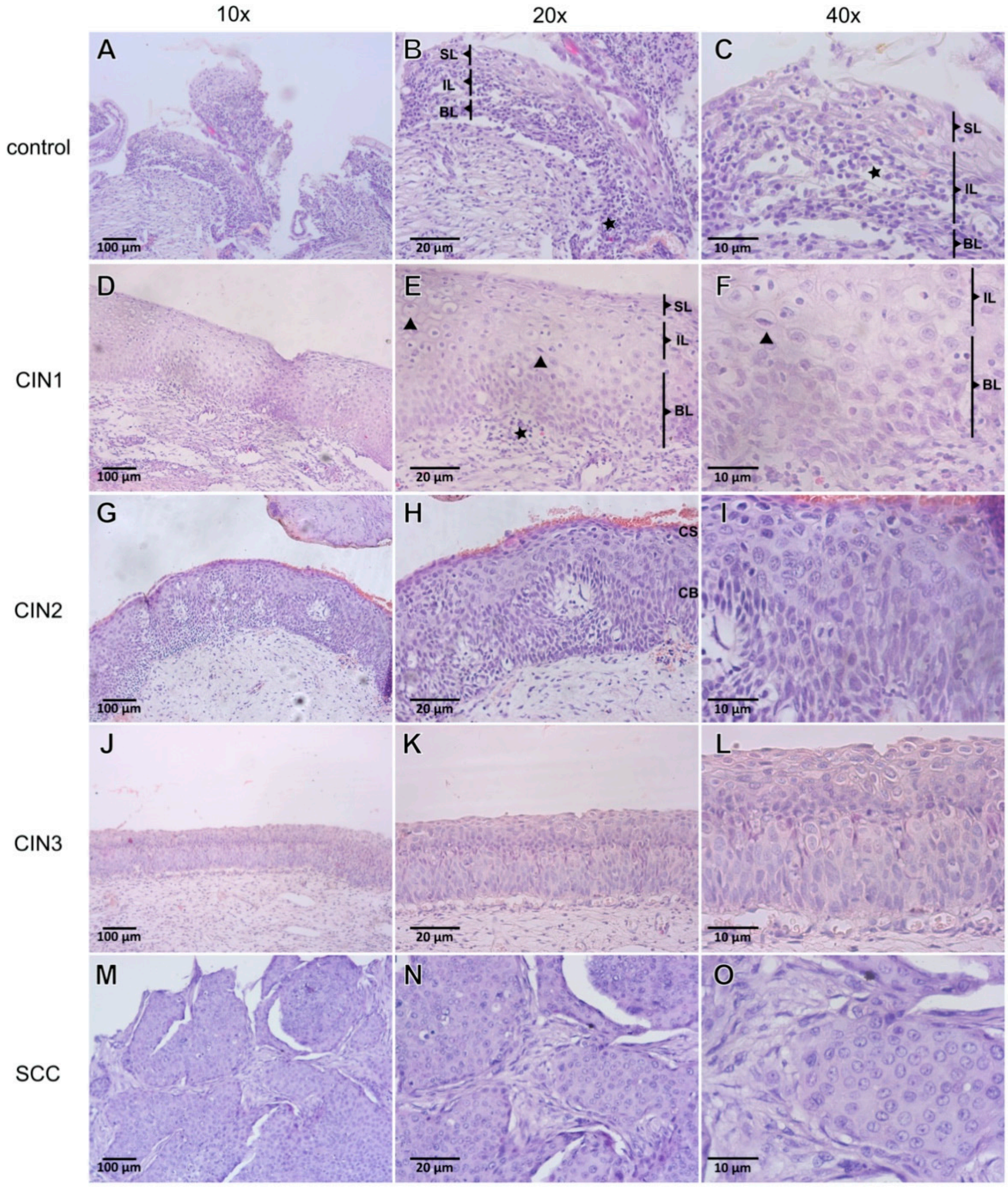

Supplementary Figure S1: *Histopathological assessment of control, cervical intraepithelial neoplasia (CIN) of grade 1 (CIN1), CIN2, CIN3 and squamous cell carcinoma (SCC) groups.* **A-C)** Control group specimens exhibited preserved superficial (SL); intermediate (IL) and basal (BL) layers, with signs of mild inflammatory infiltration (asterisk), consistent with cervicitis. **D-F)** CIN1 specimens exhibited cellular alterations present in the first third of the epithelium basal layer, with disturbances in maturation and increased nuclei size. The intermediate and superficial layers were enriched with koilocytes, characterized by irregular nuclear membranes surrounded by a perinuclear cytoplasmic halo with sharped edges (arrowhead). **G-I)** CIN2 histological sections presented maturation disturbance with increased cell nuclei size in the lower two-thirds of the epithelium, as well as the presence of koilocytes (arrowhead) and mitotic activity (arrow) in the intermediate and superficial layers. **J-L).** The CIN3 group presented maturation disorders and nuclear alterations as increased size and polarity loss of the entire squamous epithelium, as well as the presence of mitotic activity (arrow) in non-basal upper layers. **M-O)** The SCC group exhibited epithelial cells with nuclear alterations, an increase in size and loss of polarity observed in the entire lesion which formed blocks and cords invading the adjacent connective tissue.

Supplementary Table S1: Immunoreactive scoring (IRS) of βA-activin and follistatin.

**A:** Percentage of positive cells	**B:** Intensity of staining	**IRS:** multiplication of **A** and **B**
**0:** 0% positive cells	**0:** Negative reaction	**0-1:** negative
**1:** <10% positive cells	**1:** mild reaction	**2-3:** mild
**2:** 10-50% positive cells	**2:** moderate reaction	**4-8:** moderate
**3:** 51-80% positive cells	**3:** strong reaction	**9-12:** strong
**4:** > 80% positive cells	**4:** very strong reaction	**13-16:** very strong

Final IRS of βA-activin and follistatin was calculated by multiplying the percentage of positive cells (**A**) by the intensity of the positively stained area (**B**); IRS ranged from 0 to 16 score.

Supplementary Table S2: PCR protocols and conditions used for the detection of DNA-HPV

**Primers**	**PCR protocol**	**PCR conditions**	
PC03<br>PC04	1μl extracted DNA<br>+<br>9μl PCR mix: 0.5 μl of each oligonucleotid primer (10 pmol/μl), 0.2 μl Taq DNA polimerase (Phoneutria-PHT) (5 UI/ μl); 0.8 μl of dNTP's (2.5 mM); 1 μl of buffer PHT-IB (10X) and 6 μl of bidistilled water.	1 cycle:	95°C/4 mins
		29 cycles:	55°C/1 min<br>72°C/2 mins<br>95°C/1 min
		1 cycle:	55°C/1 min<br>72°C/2 mins<br>4°C/infinite
MY09<br>MY11	1μl extracted DNA<br>+<br>9μl PCR mix: 1μl of each oligonucleotid primer (10 pmol/μl) , 0.125 μl Taq DNA polimerase (Phoneutria-PHT) (5 UI/ μl); 0.8 μl of dNTP's (2.5 mM); 0.4 μl of Mg (25 mM); 1 μl of buffer PHT (10X) IB and 4.7 μl of bidistilled water.	1 cycle:	95°C/4 mins
		40 cycles:	55°C/1 min<br>72°C/2 mins<br>95°C/1 min
		1 cycle:	55°C/1 min<br>72°C/2 mins<br>4°C/10 mins
GP5+<br>GP6+	1μl extracted DNA<br>+<br>9μl PCR mix: 1μl of each oligonucleotid primer (10 pmol/μl), 0.2 μl Taq DNA polimerase (Phoneutria-PHT) (5 UI/ μl); 1 μl of dNTP's (2.5 mM); 1 μl of buffer PHT (10X) IB and 4.8 μl of bidistilled water.	1 cycle:	95°C/4 mins
		39 cycles:	95°C/1 min<br>45°C/2 mins<br>72°C/1 min
		1 cycle:	45°C/2 min<br>72°C/5 mins<br>4°C/10 mins

HPV: human papillomavirus; **dNTP**: deoxyribonucleotides phosphate

Supplementary Table S3: Nucleotide sequences of Primers used for PCR detection of DNA-HPV

**Primer**	**Target**	**Nucleotide sequences**	**Product size**	**Reference**
MY11	L1	5′ GCM CAG GGW CAT AAY AAT GG 3′	450 pb	[47]
MY09	L1	5′ CGT CCM ARR GGA WAC TGA TC 3′	450 pb	[47]
GP5+	L1	5′ TTT GTT ACT GTG GTA GAT ACT AC C 3′	150 pb	[47]
GP6+	L1	5′ GAA AAA TAA ACT GTA AAT CAT ATT C 3′	150 pb	[47]

Supplementary Table S4: Nucleotide sequence of general and type specific primers used for genotyping high-risk HPVs

Primer	Target	Nucleotide sequences	HPV	Product size	Reference
E6CF4	*E6/E7*	5'-ATT CTG TGT ATG GAG AAA CAT TAG AA-3'	HPV-DNA	306 -343	[48]
E7CR3	*E6/E7*	5'-TGA GCT GTC GCT TAA TTG CTC-3'	HPV-DNA	306 -343	[48]
16SF1	*E6/E7*	5'-TGT ATG TCT TGT TGC AGA TCA TCA-3'	HPV16	149	[48]
18SF2	*E6/E7*	5'-CCA TTC GTG CTG CAA CCG-3'	HPV18	177	[48]
31SF1	*E6/E7*	5'-GTA TGG AAC AAC ATT AGA AAA ATT GAC-3'	HPV31	300	[48]
33F<br>*33R*	*E1/E2*	5′-ATG ATA GAT GAT GTA ACG CC-3′<br>5′-GCA CAC TCC ATG CGT ATC AG-3′	HPV33	455	[49]
35F<br>*35R*	*E6/E7*	5′-CAA CGA GGT AGA AGA AAG CAT C-3′<br>5′-CCG ACC TGT CCA CCG TCC ACC G-3′	HPV35	358	[50]
52SF2	*E6/E7*	5'-CTA TTA GAT GTA TGA TTT GTC AAA CG-3'	HPV52	249	[48]
58SF1	*E6/E7*	5′- ATG TAA AGT GTG CTT ACG ATT GC-3'	HPV58	375	[48]

Supplementary Table S5: PCR protocols and conditions used for the high-risk HPVs genotyping by PCR

**Primers**	**PCR protocol**	**PCR conditions**	
E6CF4 (forward) *E7CR3* (reverse)	1μl extracted DNA<br>+<br>9μl PCR mix: 1μl of each oligonucleotid primer (10 pmol/μl) , 0.2 μl Taq DNA polimerase (Phoneutria-PHT) (5 UI/ μl); 0.8 μl of dNTP's (2.5 mM); 1 μl of buffer PHT 1B (Phoneutria) (10X) and 5 μl of bidistilled water.	1 cycle:	95°C/5 mins
		39 cycles:	HPV31: 50°C/1min HPV52: 48°C/1 min HPV58: 55°C/1 min<br>72°C/2 mins<br>95°C/1 min
		1 cycle:	HPV31: 50°C/1min HPV52: 48°C/1 min HPV58: 55°C/1 min<br>72°C/10 mins<br>4°C/10 mins
E7CR3 (reverse) *16SF1* (forward) *18SF2* (forward)	1μl of first amplification product of 450 pb<br>+<br>9μl mix: 1 μl of each oligonucleotid primer (10 pmol/μl), 0.2 μl Taq DNA polimerase (PHT, Phoneutria) (5 UI/ μl); 0.8 μl of dNTP's (2.5 mM); 1 μl of buffer PHT 1B (Phoneutria) (10X) and 5 μl of bidistilled water.	1 cycle:	95°C/5 mins
		39 cycles:	HPV16: 54°C/1min HPV18:52°C/1 min<br>72°C/2 mins<br>95°C/1 min
		1 cycle:	HPV16: 54°C/1 min HPV18: 52°C/1 min<br>72°C/10 mins<br>4°C/10 mins
	1μl extracted DNA	1 cycle:	95°C/5 mins

E7CR3 (reverse)<br>*31SF1* (forward)<br>*52SF2* (forward)<br>*58SF1* (forward)	+<br>9µl PCR mix: 1µl of each oligonucleotid primer (10 pmol/µl) , 0.2 µl Taq DNA polimerase (Phoneutria-PHT) (5 UI/ µl); 0.8 µl of dNTP's (2.5 mM); 1 µl of buffer PHT 1B (Phoneutria) (10X) and 5 µl of bidistilled water.	39 cycles:	HPV31: 50°C/1min HPV52: 48°C/1 min HPV58: 55°C/1 min<br>72°C/2 mins<br>95°C/1 min
		1 cycle:	HPV31: 50°C/1min HPV52: 48°C/1 min<br>HPV58: 55°C/1 min<br>72°C/10 mins<br>4°C/10 mins
33F (forward)<br>*33R* (reverse)	1µl extracted DNA<br>+<br>9µl PCR mix: 0.5 µl of each oligonucleotid primer (10 pmol/µl), 0.2 µl Taq DNA polimerase (Phoneutria-PHT) (5 UI/ µl); 0.8 µl of dNTP's (2.5 mM); 1 µl of buffer PHT (10X) IB and 6 µl of bidistilled water.	1 cycle:	95°C/4 mins
		4 cycles:	94°C/30 secs<br>57°C/30 secs<br>72°C/1 min
		34 cycles:	94°C/30 secs<br>55°C/30 secs<br>72°C/1 min
		1 cycle:	72°C/15 min<br>4°C/10 mins
35F (forward)<br>*35R* (reverse)	1µl extracted DNA<br>+<br>9µl PCR mix: 1 µl of each oligonucleotid primer (10 pmol/µl), 0.2 µl Taq DNA polimerase (Phoneutria-PHT) (5 UI/ µl); 0.8 µl of dNTP's (2.5 mM); 1 µl of buffer PHT (10X) IB and 5 µl of bidistilled water.	1 cycle:	94°C/20 secs
		30 cycles:	54°C/2 mins<br>72°C/1 min<br>90°C/30 secs
		1 cycle:	54°C/2 mins<br>72°C/10 mins<br>4°C/10 mins

Supplementary Table S6: Age range of the patients included in the study

**Variable**	**Control**		**CIN 1**		**CIN 2**		**CIN 3**		**SCC**		**Total**	
**Age range**	#	%	#	%	#	%	#	%	#	%	#	%
**17-35**	9	60.0%	26	68.4%	14	37.8%	5	12.8%	5	15.2%	59	36.4%
**36-50**	2	13.3%	6	15.8%	9	24.3%	8	20.5%	11	33.3%	36	22.2%
**51-65**	4	26.7%	0	0.0%	2	5.4%	1	2.6%	11	33.3%	18	11.1%
**66-80**	0	0.0%	1	2.6%	1	2.7%	0	0.0%	3	9.1%	5	3.1%
**81-90**	0	0.0%	0	0.0%	1	2.7%	0	0.0%	1	3.0%	2	1.2%

CIN: Cervical Intraepithelial Neoplasia; SCC: Squamous Cervical Carcinoma
